# Research Progress of a Pesticide Polymer-Controlled Release System Based on Polysaccharides

**DOI:** 10.3390/polym15132810

**Published:** 2023-06-25

**Authors:** Zan Zhang, Ni Yang, Jie Yu, Shuo Jin, Guangmao Shen, Hanqiu Chen, Nima Yuzhen, Dong Xiang, Kun Qian

**Affiliations:** 1 College of Plant Protection, Southwest University, Chongqing 400715, Chinayujiewildfire@163.com (J.Y.);; 2 Institute of Vegetable, Tibet Academy of Agricultural and Animal Husbandry Sciences, Lhasa 850032, China

**Keywords:** research progress, pesticide, polymer, controlled release system, polysaccharide

## Abstract

In recent years, with the development of the nanomaterials discipline, many new pesticide drug-carrying systems—such as pesticide nano-metal particles, nano-metal oxides, and other drug-carrying materials—had been developed and applied to pesticide formulations. Although these new drug-loading systems are relatively friendly to the environment, the direct exposure of many metal nanoparticles to the environment will inevitably lead to potential effects. In response to these problems, organic nanomaterials have been rapidly developed due to their high-quality biodegradation and biocompatibility. Most of these organic nanomaterials were mainly polysaccharide materials, such as chitosan, carboxymethyl chitosan, sodium alginate, β-cyclodextrin, cellulose, starch, guar gum, etc. Some of these materials could be used to carry inorganic materials to develop a temperature- or pH-sensitive pesticide drug delivery system. Herein, the pesticide drug-carrying system developed based on polysaccharide materials, such as chitosan, was referred to as the pesticide polymer drug-carrying system based on polysaccharide materials. This kind of drug-loading system could be used to protect the pesticide molecules from harsh environments, such as pH, light, temperature, etc., and was used to develop the function of a sustained release, targeted release of pesticides in the intestine of insects, and achieve the goal of precise application, reduction, and efficiency of pesticides. In this review, the recent progress in the field of polysaccharide-based polymer drug delivery systems for pesticides has been discussed, and suggestions for future development were proposed based on the current situation.

## 1. Introduction

With the widespread use of pesticides, the active ingredients of traditional pesticide forms were released into the field immediately upon application. However, various physical factors in the natural environment, including light, water, soil, and microorganisms, can degrade, absorb, and migrate the active components of pesticides, leading to a rapid reduction in their concentration and effectiveness against harmful organisms. Unfortunately, the overuse of pesticides has resulted in pesticide residues in the environment, and some ecological preventive and control measures have proven ineffective in combating pests.

To tackle the challenges of pesticide residues, pest resistance, and pest resurgence, it is important to optimize existing pesticide formulations using technology or equipment. In recent years, emerging pesticide nanotechnology sustained-release technology has proven to be a promising solution for these issues. This technology allows for the release of pesticides at regular intervals with a constant concentration, reducing the volume of pesticides necessary while still maintaining their efficiency.

Moreover, with the advancement of nanomaterials, some new drug-carrying systems using nano-metal particles or oxides have been developed. These advancements have significantly optimized pesticide preparations, enhancing the efficacy of controlling agricultural pests while minimizing the impact of pesticide residues on the environment. However, a large number of nano-metal particles has been exposed to the environment, which might cause harm to the environment and crop [1], such as the nano-silver particles—which have been studied in recent years as a pest control [2]—and other pesticide systems with metal particles or oxides as carriers [3]. In addition, metal nanoparticles exposed in the field might also be dispersed in the air, causing secondary air pollution [4]. Therefore, the study of natural polymer materials as pesticide polymer-controlled release carriers has become a new hot spot. A new sustainable pesticide release system has been developed using nanotechnology and multiple carbohydrates as raw materials. This system regulates the release rate and time of pesticides, improving their efficiency and environmental performance while providing better pest protection. Compared to traditional chemical pesticides, this system is safer and more environmentally friendly.

**Figure 1:**
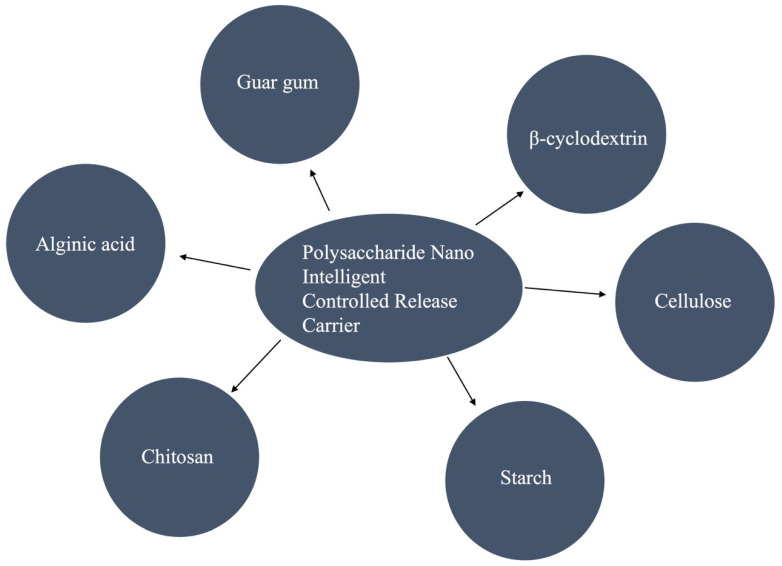
Categories of a polysaccharide nano-intelligent-controlled release carrier.

Polysaccharides are macromolecules composed of monosaccharides linked by glycoside bonds. These compounds are mainly natural organisms and are widely distributed in nature, such as in animals (chitosan, CS), plants (cellulose, pectin guar gum, starch and β-cyclodextrin), microorganisms (glucan), algae (alginate), and other natural products, which are cheap to obtain and easy to produce (Figure 1). Polysaccharide nanomaterials are used as carriers in pesticide preparations. They can control the concentration of active ingredients of pesticides, prevent the decomposition, volatilization and loss of active ingredients, and they can also prolong the duration of drugs and solve the environmental problems caused by pesticide additives. The polymer-controlled release system formed by the carrier made of natural polysaccharide materials is called a polymer-controlled release system and is based on natural polysaccharides. In addition, to make up for the shortcomings of the controlled release of natural materials, based on natural polysaccharide materials, polysaccharide derivatives are formed through chemical synthesis. The polymer-controlled release system, which takes polysaccharide derivatives as the carrier, is called the polymer-controlled release system based on the polysaccharide derivatives.

Here, we summarize the development of pesticide nano-controlled release drug delivery systems based on polysaccharide materials in recent years. The pesticide delivery system developed using polysaccharide materials, such as chitosan, is called a polysaccharide-based pesticide polymer drug delivery system. This drug loading system can protect pesticide molecules from adverse environmental factors, such as pH value, light, temperature, etc., and achieve a continuous release and precise application of pesticides, as well as achieve target release in insect intestines, thus reducing pesticide use and improving efficiency. Polysaccharide materials are naturally occurring biomaterials that have the advantages of environmental friendliness, naturalness, and safety properties. At the same time, the structure and function of polysaccharide materials can be modified and adjusted through biotechnology, thus forming a variety of polysaccharide carriers with specific target and delivery properties. Currently, polysaccharide materials as pesticide carrier materials have attracted widespread attention as a research hotspot. In this review, we will discuss the latest progress of polysaccharide-based polymer drug delivery systems in the field of pesticide delivery. In addition, we will point out some challenges and future directions for this field. Due to the inconsistency between symbols and noun abbreviations used in many citations, please refer to Table S1 for abbreviations covered in this article.

## 2. Research Progress of Natural Polysaccharide Nano-Formulations of Pesticides

### 2.1. Research Progress of Chitosan and Its Derivatives as Nanoscale Sustained and Controlled Release Carriers

Among natural polysaccharide materials, chitosan is obtained by the deacetylation of chitin and was a polysaccharide biopolymer. Chitin is naturally present in the cuticle of crustacean insects and in the cell walls of most fungi and algae. Additionally, chitosan has been favored by many researchers due to its convenience, especially its good biological function, body compatibility, and microbial degradability, and has been used in many industries. At present, many researchers have used chitosan as a controlled release substrate for a drug-sustained release (Table 1, Figure S1). At the same time, chitosan has been widely used in pesticide-controlled release preparations due to its own characteristics and insecticidal and antibacterial activities [5,6].

In recent years, chitosan has been used as the carrier for the pesticide control system used to prepare nano-controlled release carriers in the following ways. The first one is to directly use pure chitosan to prepare chitosan nanoparticles as the carrier of effective pesticide molecules. For example, chitosan (analytical grade) was directly prepared into chitosan nanocomposites by an ion gel method, and secondary metabolites of *Beauveria bassiana* were used as biopesticides to effectively control *Spodoptera litura* and have no effect on the non-target organism zebrafish [28]. In addition, chitosan can also encapsulate Spinosad to make a controlled release preparation of Spinosad, an environmental-friendly biopesticide. Chitosan nanoparticles endow the dosage form with pH and temperature sensitivity and excellent UV shielding [24]. This showed that only using chitosan as the preparation material of the controlled release system carrier could also form an environmentally friendly, efficient, and low-toxic insecticidal nano-controlled release agent. The second is that chitosan can be prepared into a new type of metal nano-pesticide formulation formed by nano-gel loaded with effective pesticide molecules. For example, chitosan was prepared into a nano gel and directly loaded with copper ions to form a copper ion nano-controlled release agent, which was a copper-based pesticide with pH and a chitosan hydrolase-controlled release. At the same time, chitosan nano gel could also enhance copper ions to inhibit the growth of *Fusarium graminearum* [9]. The third kind of chitosan is loaded with other compounds to form a nanocomposite film and then loaded with active ingredients of pesticides to prolong the effective time of the pesticides. For example, after chitosan, vinyl acetate and ethylene copolymer constructed micellar nanocomposite film loaded with fungicide Iprodione, it showed a better antifungal ability and temperature-sensitive drug release performance [23].

The chitosan composite nanoparticles are prepared by mixing chitosan with other compounds, and specific compounds are selected to prepare chitosan composite nanoparticles to make up for the shortcomings of the active ingredients of pesticides, enhance the advantages, improve the efficacy, and prolong the duration of the drug. For example, a new type of nanoparticle prepared by mixing pectin, chitosan, and sodium tripolyphosphate (PEC/CS/TPP) could carry the active ingredient of paraquat to form a paraquat nano-controlled release agent. Such nanoparticles could not only enhance the herbicidal activity of paraquat on corn or mustard, but also effectively reduce the adsorption of paraquat to the soil, thereby reducing the pesticide residue of paraquat. At the same time, it could also reduce the toxicity of paraquat to the lungs of mammals such as rats [18,19]. The nanopesticide system was prepared by the shell polysaccharide and alginate, and also reduced the concentration of paraquat released in the soil and the pollution of surface and underground hydrological systems [7]. The similar nanopesticide system of chitosan/tripolyphosphate nanoparticles could also reduce the adverse effects of the pesticide nicotine hydrochloride (NCT) on human health [14]. Mixing chitosan with other compounds to prepare chitosan composite nanoparticles can not only reduce the toxicity of effective pesticide molecules to non-target organisms and environmental pollution, but also protected the active ingredients of pesticides, avoid their degradation, and prolong the release time. For example, the high volatility and chemical instability of citral limited its application in agricultural production, and the use of the copolymer chitosan/carboxymethylcellulose (CS/CMC) hydrogel microspheres to load citral can avoid the active ingredient volatility and increase stability [25]. The chitosan oligomer (CO) was used as membrane material, and diphenylmethane-4,4′-diisocyanate (MDI) was used as a cross-linking agent to prepare polyurea microencapsulated abamectin (AVM), which could not only maintain AVM, but also extended the release time (up to 120 h) and reduce the degradation of AVM significantly without residue [21]. In addition, chitosan (CS) can also encapsulate AVM with nanoparticles composed of poly-γ-glutamic acid (γ-PGA), reducing its photolysis, and providing the function of an alkaline-controlled release [15]. After the fungicide hydrophobic hexaconazole was loaded on chitosan nanoparticles prepared by the cross-linking agent sodium tripolyphosphate (TPP), the fungicide in the nanoparticles was released in a sustainable manner for up to 86 h. Antifungal studies in vitro confirmed that the smaller particle size of nano formulations enhanced the antifungal activity against *G. boninense* [20].

Chitosan can also select corresponding compounds according to the different physical and chemical properties of the active ingredients of pesticides and mix them to prepare specific chitosan composite nanoparticles to improve the loading rate of active ingredients of pesticides.

For improving the loading rate of the active ingredients of pesticides, based on the different physical and chemical properties of the active ingredients of pesticides, corresponding compounds were selected and prepared with chitosan. For example, chitosan, starch, and alginate were prepared as composite nanoparticles to encapsulate and control the release of spirotetramat [26]. Chitosan-poly(lactide) copolymers could carry lipophilic imidacloprid [8], and the chitosan–gelatin microspheres could carry the neonicotinoid dinotefuran (DIN) [27].

In addition to directly using chitosan as a substrate, chitosan derivatives were obtained by adding new functional groups for modification so that chitosan could obtain new properties, so as to bridge the shortcomings of drugs and meet the needs of intelligently controlled release systems. For example, amphiphilic biocopolymers synthesized by adding carboxymethyl groups to chitosan and then mixing them with aqueous azidobenzaldehyde (Az) [11]. The amphiphilic chitosan derivative, *N*,*N*-dimethylhexadecylcarboxymethyl chitosan (DCMC), was used as a carrier for rotenone [13]. Hydroxyisopropyl chitosan (HPCTS, positively charged) and carboxymethyl chitosan (CMC, good water solubility) were successfully used as degradable water-soluble carriers of AIEAS to achieve its controlled release [22], and so on. Furthermore, a novel nanocomposite (cl-Ch-pMAc@ZnO/CdSQDs) was developed under microwave irradiation by preparing ZnO/CdS QDs on anionically functionalized chitosan. It had anti-photodegradation and antibacterial activity, with the biggest feature being that it could be regenerated by changing the pH and be reused [17].

### 2.2. Research Progress of Cellulose and Its Derivatives as Nanoscale Sustained and Controlled Release Carriers

Cellulose molecules are composed of β-D-glucopyranose units linked by β-(1,4) glycosidic chemical bonds. Hemicellulose, on the other hand, is a heterogeneous polymer of several different types of monosaccharides similar to cellulose. Cellulose and hemicellulose are abundant in plants and have good biocompatibility, low toxicity, and natural biodegradability. In recent years, it was often used as a carrier for active ingredients of pesticides, prepared into environmentally friendly nanoparticle materials, and coated with active ingredients of pesticides to form a controlled release agent for pesticides (Table 2, Figure S2).

Due to their long polysaccharide chains, cellulose and hemicellulose are generally prepared by interacting with crosslinking agents to form a composite network polymer, which increased the stability of nano dosage forms and the loading rate and release rate of active ingredients of pesticides. For example, for increasing the specificity and sensitivity of gel particles to release drugs at pH [34], cellulose was added to enhance the gel polymerization ability in preparing gelatin nanopesticides. Among them, cellulose and hemicellulose could be degraded by specific enzymes, so they could also be prepared into nanomedicines with specific targets and a controlled release according to this characteristic. For example, xylan, which is naturally present in plant cell walls, and a toluene diisocyanate (TDI) cross-linker were used to prepare loaded xylan-based nanocarriers for the usage of fungicides. Additionally, xylan in xylan-based nanocarriers could be digested by enzymes contained in the fungus, releasing the fungicide. Therefore, xylan-based nanocarriers had the function of being degraded by fungi, which was the trigger factor of agricultural chemicals [33]. Due to their characteristics, for increasing their network complexing ability, cellulose and hemicellulose usually need to add other functional groups to produce cellulose and hemicellulose derivatives, which are then used to prepare nano-controlled release agents.

In some cases, some properties of natural products, such as cellulose and hemicellulose, could not meet the conditions as a carrier. Therefore, chemical modifications were used to enhance the function of polysaccharide materials to meet the increasing number of pesticides. For example, to improve the adhesion and anti-ultraviolet properties of fibers and achieve the purpose of optimizing pesticide loading and intelligent controlled release, chemical means were used to combine carboxymethyl with cellulose [25,32,35].

In addition, researchers had added hydroxyethyl functional groups to cellulose to meet the loading of tebuconazole and an intelligent controlled release. There was also the addition of ethyl functional groups to cellulose, which reduced the mobility of the technical compound in soil and protects it from photodegradation. In addition, a pH/cellulase dual-stimuli-responsive controlled release formulation was designed by grafting hydroxypropyl cellulose onto pyraclostrobin-loaded hollow mesoporous silica nanoparticles using ester bonds [36], and the composite polymer network was formed with cellulose ester MCN to improve the pesticide effect and bioavailability of drugs [29,30].

### 2.3. Research Progress of Starch, Dextrin and Their Derivatives as Nanoscale Sustained and Controlled Release Carriers

Starch was mainly found in crop products and was its main energy storage compound, mainly composed of amylose and amylopectin. Both amylose and amylopectin were polysaccharides composed of glucose units but connected by different glycosidic bonds. Amylose was a direct α-(1,4) glycosidic bond, while amylopectin, in addition to the same main chain as amylose, also contained a branched chain structure formed by α-(1,6) glycosidic bonds. In nature, these two starches were naturally mixed and may vary within the same species, depending on the maturity of the plant [37,38,39]. Starch was also a long-chain polysaccharide molecule, like cellulose and guar gum. It was used in nano-controlled release agents in the state of network complexes and was mostly prepared as a herbicide sustained-release drug-carrying system (Table 3, Figure S3). For example, starch and polyvinyl alcohol composite materials could be used as a drug-loading system to carry the herbicide atrazine (AT) to prepare a herbicide-controlled release system, which could reduce the leaching of the herbicide from the soil layer and the pollution of pesticides to the environment [40]. In addition, starch could also be prepared into composite gel beads with chitosan, and calcium alginate to increase the degree of complexation of chitosan and calcium alginate, for reducing the release rate of the active ingredients of the loaded pesticide, and prolonging the duration of the drug [26]. Similarly, starch generally needed to be added with other functional groups to make starch derivatives increase different characteristics and network complexing ability, and then used to prepare nano-controlled release agents.

Cyclodextrin is a general term for cyclic oligosaccharides formed by 6 or more α-D-glucopyranose. It exists naturally in plants, such as aloe, and could also be produced from starch by cyclodextrin glucotransferase. Among them, the common cyclodextrins mainly include α-cyclodextrin (consisting of six glucose units), β-cyclodextrin (seven units) and γ-cyclodextrin (eight units) [47,48]. Cyclodextrins are carriers of natural hydrophobic pesticide active ingredients due to their unique slightly tapered hollow cylindrical ring structure, hydrophilic outer edge, and hydrophobic inner cavity. Initially, most of the loads carried by cyclodextrin were organophosphorus pesticides, such as malathion, dichlorvos, cymetrate, chlorpyrifos, sulfaphos, etc. Through the encapsulation of cyclodextrin, the characteristics of such pesticides as fluidity, wettability, and thermal stability could be improved and remove the volatilization characteristics of organophosphorus pesticides [49]. The compound formed by wrapping carvacrol (CVC) or linalool (LNL) with β-cyclodextrin overcome the problems of low water solubility, high photosensitivity and high volatility of carvacrol and linalool [45]. In addition, some researchers linked cyclodextrin building blocks and used the sponge-like structure as a nano-controlled release carrier system for the long-term release of herbicides [41,46]. With the research on the characteristics of cyclodextrin, in recent years, cyclodextrin has been widely used to recover the pesticide pollution remaining in the environment. Additionally, artificially modified starch derivatives, under the optimization of additional functional groups, could have a better loading rate and excellent characteristics, such as carboxymethyl starch and CMS composite system from amylopectin for herbicides; namely Isoproturon was encapsulated (about 75% encapsulation rate), and the herbicide release rate in water was significantly reduced [43].

### 2.4. Research Progress of Alginic Acid and Its Derivatives as Nanoscale Sustained and Controlled Release Carriers

Alginic acid is a polysaccharide naturally present in the cell wall of brown algae, which is formed by the linear polymerization of mono-uronic acid. Alginic acid was easy to form a gel with cations, and it further formed a nanostructure to be used as a drug-release matrix. The nano-controlled release system formed by alginate was generally prepared into alginate nanoparticles, which were coated or absorbed with pesticide active ingredients to form pesticide-controlled release nano dosage forms. Pesticide nano-controlled release agents could intelligently control the release time or release concentration of pesticides according to environmental characteristics (Table 4, Figure S4).

Similarly, alginate was also one of the materials that were often used in the preparation of polysaccharide nano-controlled release systems in recent years. For example, the alginate/chitosan nano-pesticide system was prepared according to the highly toxic properties of paraquat, which changed the release profile of the herbicide and its interaction with the soil, reducing the negative impact of paraquat [7]. According to the high solubility and volatility of the herbicide active ingredient dicamba, which had the disadvantage of strong surface water and groundwater and atmospheric loss, the coating of alginate nanoparticles was used to make up for its disadvantages [57]. In addition, a novel esterase/glutathione (GSH)-responsive photoactivatable nanopesticide delivery system synthesized using sodium alginate (SA) as a substrate and effectively protected conjugated phloxine B from photodegradation in vitro [55]. The polysaccharide nano-controlled release system prepared by alginate could not only control the diffusion of active ingredients of pesticides and avoid pesticide pollution, but also could be prepared as a slow-release pesticide loading system. For example, sodium alginate and chitosan were prepared as herbicide grass of a glyphosate-controlled release material and made glyphosate release continuously within 30 days [58]. The alginate–lentinan–amino-oligosaccharide hydrogel (ALA-hydrogel), prepared by electrostatic interaction using alginate–lentinan, not only had a stable sustained-release activity, but also could continuously and strongly induced plant resistance to TMV and increased the release of calcium ions to promote the growth of *Nicotiana benthamiana* [53]. Gelatin beads made from alginate could be loaded with pheromones (lauryl acetate), and slowly released the pheromones into the atmosphere over a long period of time [50]. The intelligent controlled release against agricultural pests was generally based on the fact that the prepared nano-controlled release formulation was sensitive to pH, temperature, or light, and quickly released the active ingredients of pesticides to kill agricultural pests in a specific biological living environment. For example, sodium alginate and gelatin biopolymer beads were prepared using CaCl_2_ as a crosslinker to load the insecticide cypermethrin. The beads could control the insecticide release by temperature and pH [51]. Using the helix–helix structure transformation of gelatin, the polypyrrole/calcium alginate/gelatin (PPy/calcium alginate/gel) drug delivery system could be loaded with carbendazim to form a light-controlled release system [56]. A pH-controlled release system loaded with tetramycin could be prepared by a pH-sensitive oxidized alginate-based double-crosslinked gel [54].

To artificially control the release rate of acetamiprid, cholesterol-grafted sodium alginate derivatives (CSAD) of different molecular weights synthesized by esterification were used as carriers [52].

### 2.5. Research Progress of Other Polysaccharide Materials and Their Derivatives as Nanoscale Sustained and Controlled Release Carriers

In addition to the biological polysaccharide materials introduced above, there were some other polysaccharide biological materials that could be used in the preparation of nano-controlled release preparations, such as gum, dextran, etc. [59]. Among them, pectin naturally existed in the cell wall of higher plants and was a linear polysaccharide composed of D-galacturonic acid units connected by α-(1,4) bonds. At present, pectin was mostly used as a matrix, combined with other compounds or metals to prepare a pectinase-sensitive pesticide-controlled release system. For example, by combining pectin with metals, a smart redox and pectinase dual stimuli-responsive pesticide delivery system was constructed [60]. The preparation of pectin and cedarwood essential oil into nanocapsules could carry the insecticidal efficiency of insecticides and avoid the residues of insecticides in the environment [61]. Dextran mostly existed naturally in bacteria, and its structure was mainly composed of the main chain composed of glucose monomers through α-(1,6) bonds, and branches composed of glucose monomers through α-(1,4), α-(1,3), and α-(1,2) [62,63]. Compared with other polysaccharides, the main chain structure of dextran was relatively changeable, and the most notable feature was its insolubility in water. This feature could encapsulate water-insoluble pesticide analysis, such as pyraclostrobin (PYR), and further prepare pH-sensitive acetalized dextran microparticles (Pyr@Ac-Dex) [14]. In addition, based on the insoluble characteristics of dextran in water, it was often transformed into a matrix for collecting organophosphorus pesticide residues, such as using dextran to enhance the organophosphorus detection ability of pesticide residue analysis instruments [64,65].

Guar gum is a non-ionic, water-soluble natural polysaccharide that can generally be extracted from the seeds of the leguminous plant *Cyamopsis tetragonoloba*. It uses polymannose as the molecular backbone—D-mannopyranose units were connected by β-1,4 glycosidic bonds, and D-galactopyranose was connected to polymannose by α-1,6 glycosidic bonds. Microorganisms in the gastrointestinal tract can partially ferment polymannose to produce a large number of short-chain fatty acids. Guar gum is a main chain with a multi-branched structure, which is easy to form a network complex, and then load the active ingredients of pesticides to form a capsule sustained-release structure (Table 5, Figure S5). For example, the copolymer of guar gum, acrylic acid, and acrylamide were cross-linked with monomers of guar gum and acrylic acid into a hydrogel, which could provide a gel system for long-term drug release and conducive to the slow release of a micro-fertilizer system and related pheromones [66]. In addition, for herbicides that require long-term release, it was more appropriate to use pectin and guar gum to make herbicide delivery systems. It had been found that carfentrazone-ethyl and guar gum were prepared into a polymer-herbicide conjugate gel formulation, which could be used for the long-term release of herbicide active ingredients. Diffusion of the active ingredient in the polymer-herbicide conjugate resulted in greater coverage (most weed leaf stomata (>95%)) compared to conventional spraying. In addition, the weed mortality of Anagallis arvensis (95.4%), Chenopodium album (~97%), and Ageratum conyzoides (93.16%) treated with the polymer was significantly increased [67]. Using a guar gum-g-cl-polyacrylate/bentonite hydrogel composite (GG-HG) and a guar gum-g-cl-PNIPAm nanohydrogel (GG-NHG) as carriers, imidazo-controlled release formulations of tobacco herbicides had a relatively long-term herbicidal effect [68]. Guar gum and diquat prepared into a gel (Hydrogel) could significantly reduce the drift of off-site herbicides [69]. In addition, guar gum could also be made into a film (GC film) with related repellents, which was both effective and environmentally friendly and could slowly release the brown planthopper repellent citral [70].

## 3. Outlook

Polysaccharides and their derivatives have good biocompatibility and would not directly cause harm to crops and the environment. In addition, due to its biodegradable properties, it would not remain in nature for a long time to cause residual pollution. The above characteristics lead to polysaccharides and their derivatives as the preferred pesticide carrier for sustainable agriculture. In the previous report, partially nano-sized polysaccharide materials could not only inhibit the damage of harmful organisms, but also directly act on crops to stimulate the crops’ resistance. In addition, some reports found that nano polysaccharides could regulate the growth of plants themselves.

**Figure 2:**
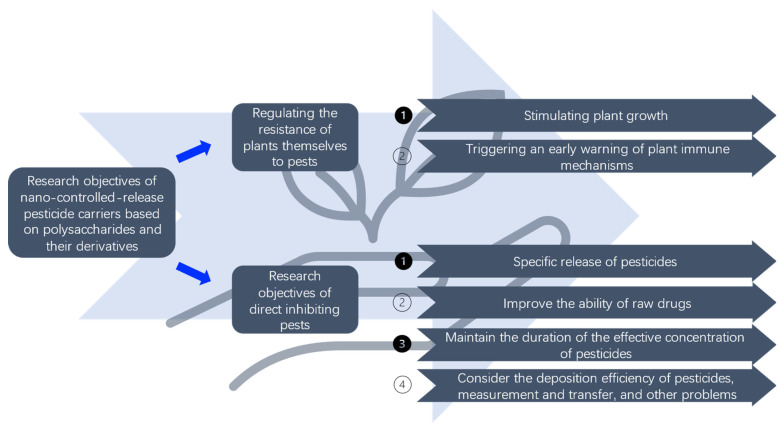
Research objectives of the nano-controlled release pesticide carriers based on polysaccharides and their derivatives.

Based on this, we speculated that nano-controlled release pesticide carriers, based on polysaccharides and their derivatives, could be developed simultaneously from the direction of regulating the resistance of plants themselves to pests and directly inhibiting pests (Figure 2). From the direction of regulating the resistance of plants themselves to pests, such as stimulating plant growth [31,70,71,72,73], triggering an early warning of plant immune mechanism [53,74]. From the direction of direct inhibiting pests, the first development direction is the specific release of pesticides, which is based on the location of pests and diseases and the period of harm, such as targeting specific enzymes’ environments in harmful organisms [9,33], or targeting specific pH [9,15,24,27,29,32,34,35,36,52,53,54,55] or temperature [23,24,27,51,53,56] environments, while protecting other species, such as bees, and earthworms have a poor release capacity in vivo. It can also improve the ability of raw drugs to be applied in the actual production process and protect the active ingredients of pesticides that are easy to decompose under light, high temperature, and pH conditions [14,15,17,21,25,32,35,41,45,66]. In addition, the duration of the effective concentration of pesticides is maintained. The release of pesticides is reduced by a low to high, and only when the concentration reaches a certain height can the harmful organisms be killed. The controlled release vehicle maintains the drug concentration in the environment as much as possible above the effective concentration [7,8,18,19,20,22,26,30,35,40,43,46,50,52,57,66,67,68]. Finally, it is necessary to consider the deposition efficiency of pesticides, measurement and transfer, and other problems in the application process [8,11,13,22,26,29,55].

In addition, because the characteristics of the polysaccharide material might not be sufficient to carry the corresponding original drug molecules, the development of polysaccharide derivatives could not only make up for this shortcoming, but also link the corresponding trigger groups, such as photolysis, enzymatic hydrolysis, redox responsive degradation, pH-controlled release [75], etc., so as to further develop a variety of intelligently controlled release carriers to achieve the targeted drug release and protection. The polysaccharide derivatives produced by adding various groups had broad application scenarios and were safer and smarter. Moreover, it was possible to combine multiple polysaccharides together and fully exploit their individual characteristics to achieve multiple functions. Different types of polysaccharides possessed distinct properties and functionalities. For example, certain polysaccharides exhibited excellent drug encapsulation capabilities (e.g., cyclodextrin, starch), while others had an exceptional stability and controlled release properties (e.g., alginates). By combining these polysaccharides, it was possible to construct carrier systems with multiple properties, thereby meeting the requirements of different drugs and aligning more closely with the concept of environmental protection [25,76]. The combined use of multiple polysaccharides also increased the stability and controllability of carriers, improved the sustained-release effects of drugs, and further enhanced therapeutic efficacy. Therefore, in the development of pesticide polymer-controlled release systems, the combined utilization of polysaccharides held great potential and offered more choices for environmentally friendly drug delivery systems.

## 4. Conclusions

The advantages of pesticide polymer-controlled release systems constructed using natural polysaccharides and polysaccharide derivatives as carriers were summarized in this article. The mechanisms of different carriers were discussed, and the future direction of pesticide polymer-controlled release systems was predicted. Despite the significant ecological advantages of pesticide polymer-controlled release systems constructed using natural polysaccharides and polysaccharide derivatives as carriers, as well as the increasing number of related research publications in recent years, they still face some challenges in practical production processes.

Firstly, there was no mature system that could fully load most or some of the existing pesticides. The characteristics of certain polysaccharide materials were insufficient to carry the corresponding active drug molecules. Therefore, this drawback could only be compensated by developing polysaccharide derivatives and connecting them with corresponding triggering groups (such as photodegradation, enzymatic degradation, pH-controlled release, etc.). Further development of various intelligent controlled release carriers was needed to achieve the targeted drug release and protection. This also led to an increase in the corresponding development costs. Secondly, in addition to nano-intelligent-controlled release carriers replacing the traditional toxic and environmentally polluting carriers for pesticides, more environmentally friendly and intelligent pesticides were being developed. For example, biopolymer drugs, such as insecticidal spider peptides, toxic insecticidal proteins, and lethal dsRNA, were under development. The delivery of these biopolymer drugs combined well with natural polysaccharide carriers. This was a research direction that had been reported in only a few papers and was also a research direction for natural polysaccharide nano-controlled release carriers. Furthermore, in the research on nano-controlled release agents for herbicides, many reports showed that natural nano-polysaccharide-controlled release carriers improved their weed-killing efficiency while reducing potential environmental, human health, and crop damage risks. After employing natural nano-polysaccharide-controlled release carriers to mitigate the risks to the environment and human health from previously banned pesticides, it was expected that more of these pesticides could be reintroduced into practical production. However, the development of natural nano-polysaccharide-controlled release carriers was still a lengthy process.

In short, constructing pesticide polymer-controlled release systems using natural polysaccharides and polysaccharide derivatives as carriers was in line with the sustainable development strategy of balancing the economy and environment. It was an important direction for the future development of pesticides.

## Figures and Tables

**Table 1:** Nano-intelligent-controlled release carrier system based on chitosan.

Methods	Key Features	pH Sensitivity	Temperature Sensitivity	Publish Date	Results	References
Ionotropic pre-gelation of polyanion with f chloride followed by polycationic crosslinking	Reduce the concentration of the release of Paraquat in the soil	NULL	NULL	15 June 2011	The herbicide showed good association with the alginate/chitosan nanoparticles, which altered its release profile. Sorption tests showed that the soil sorption profile was reduced when paraquat was associated with the nanoparticles, hence improving the herbicidal action.	Silva et al. [7]
86 °C with magnetic stirring in a nitrogen atmosphere	Sustained release, equipped with lipophilus	NULL	NULL	July 2011	Control of the submicron particle size, the imidacloprid loading content and the imidacloprid release behaviors can be achieved by optimizing the copolymer to imidacloprid ratio. The submicron particles can prolong the pesticide release time owing to the amphiphilic structure of the copolymer.	Li et al. [8]
Physical gelation in reverse emulsion	pH sensitivity and enzyme sensitivity (chitosanase).	The cumulative release increased with the decreased pH from 5.5	NULL	15 February 2013	The adsorption capacity of the pure chitosan nanogels was comparable to that of chitosan in solution and strongly depended on pH. The maximum adsorption capacity for copper(II) was obtained at pH 5 where 300 mg of copper(II) were bound to 1 g of chitosan.	Brunel et al. [9]
Methods reports by Yin et al. [10]	Oil-water amphiphilic carrier	NULL	NULL	10 March 2014	The release of methomyl from the various methomyl-loaded samples into an aqueous medium at pH 6.0 has been shown to be diffusion controlled. The diffusion rate or release rate was controlled by the shell cross-linking and its degree of cross-linking.	Sun et al. [11]
Methods reports by Li et al. [12]	Oil-water amphiphilic carrier	NULL	NULL	July 2016	DCMC which consists of hydrophilic segment of carboxymethyl group and hydrophobic region of hexadecyl chain was successfully synthesized and characterized.	Kamari et al. [13]
Ionic gelification method	Increase the stability of the original drug and prevent its non-target diffusion	NULL	NULL	7 February 2018	The release of pesticide nicotine was significantly underdeveloped in NPs, especially in NPs with NaCl.	Yang et al. [14]
Poly-electrolyte self-assembly method	Reduce photolysis, alkaline control interpretation	The cumulative release increased with the increasing pH from 5.5–8.5	NULL	June 2018	The AVM in the CS/γ-PGA nanoparticles was rapidly released under alkaline conditions and had good ability to resist photolysis.	Liang et al. [15]
Methods reports by Xuan et al. [16]	Prevent light degradation and antibacterial activity, by changing pH reuse use	NULL	NULL	5 May 2019	The synthesized nanocomposite has high effectivity for adsorption as well as degradation under solar light irradiation and is worth to be considered as an excellent antibacterial agent towards *E. coli* and *B. subtilis*.	Midya et al. [17]
Ionic gelification technique	The delay release of the Paraquat	NULL	NULL	22 May 2019	There was a kind of delayed release of paraquat from PEC/CS/TPP nanoparticles. There was almost no release until 30 min.	Rashidipour et al. [18,19]
Ionic gelification method	Sustained release, improve antibacterial activity	NULL	NULL	8 July 2019	Chitosan–hexaconazole nanoparticles at 5 mg/mL of TPP presented the highest loading content of 16.7% as well as the sustained release of 99.91% with a prolonged release time of hexaconazole up to 86 h. The fungicide nanodelivery system provided longer efficient time, low toxicity, and high antifungal activity towards *G. boninense* as well as low EC_50_ value.	Maluin et al. [20]
Grafting reaction	Sustained release, prevent the original drug degradation, and no residual pollution	NULL	NULL	1 November 2019	A BTDA-modified polyurea microcapsule was successfully prepared by first grafting BTDA to CO followed by emulsion interfacial polymerization between CO and MDI. The prepared microcapsules showed a sustained release of AVM for a longer period and protected the encapsulated AVM from sunlight.	Fu et al. [21]
Electrostatic attraction, self-assembly	Sustained release, water -soluble control conveying system	NULL	NULL	1 November 2020	The use of chitosan derivatives for the electrostatic encapsulation of herbicides on plants has achieved the release of herbicides. The use of chitosan as a carrier for the release of herbicides has been well controlled. The herbicides released from the chitosan carrier show good weed control effects on herbaceous plants.	Xie et al. [22]
Nano-precipitation method	Improved antifungal ability and temperature sensitive drug release	NULL	The release of pesticide increased with the increasing temperature from 4 to 40 °C	30 November 2020	By incorporating CS-reinforced EVA with ID-encapsulated PEG-PCL micelles to fabricate nanocomposite films with good permeability and controlled drug release capability. The introduction of the IPP micelles into the films obtained an improved antifungal ability (10 ± 0.5 mm) and the temperature-sensitive drug release ability (71.24%).	Xiao et al. [23]
Coprecipitation-based synchronous encapsulation	pH and temperature sensitivity and excellent ultraviolet shielding ability	The cumulative release decreased with the increasing pH from 1.2–9.0	the release of pesticide increased with the increasing temperature from 20 to 50 °C	1 December 2020	The in vitro release tests showed good pH and temperature sensitivity, high CRR (>80%) and long sustained-release time (>18 d), with the release dynamics obeying the Fickian diffusion mechanism of Ritger-Peppas model.	Li et al. [24]
the CS-citral emulsion was slowly dripped into the CMC solution under slow stirring to form microspheres.	Increase the stability of the original drug, prevent its non-target diffusion and extend the effect period	NULL	NULL	1 December 2020	Polymeric CS/CMC-based bio-composite scaffolds were successfully prepared, and CS/CMC hydrogel microspheres were loaded with citral in situ. The bioactive matrix systems serving dual functions may not only improve the antibacterial activity of CS under neutral pH conditions, but at the same time has the advantages of reducing the loss of volatile organic compounds and oxidation of citral.	Ma et al. [25]
Extrusion–exogenous gelation method	Slow release, increase the loading rate	NULL	NULL	January 2022	SCCA formulation exhibited the highest EE and DL and the highest capacity of controlled release of spirotetramat. The degradation rate of spirotetramat in the SCCA formulation was obviously slower than that of spirotetramat in the commercial formulation.	Xie et al. [26]
Spray-drying technology	pH and temperature sensitivity, reduce the loss of original drug immersion	The cumulative release increased with the increasing pH from 5.0–10.0	The release of pesticide increased with the increasing temperature from 10 to 30 °C	1 February 2022	DIN@CS-GEL enabled marked pH and temperature responsiveness, a feature suitable for the intelligent controlled release of DIN. The biodegradable composite carriers (CS-GEL) immobilized and protected the DIN in soil, thereby significantly reducing pesticide leaching.	Zhang et al. [27]

**Table 2:** Nano-intelligent-controlled release carrier system based on cellulose.

Methods	Key Features	pH Sensitivity	Temperature Sensitivity	Publish Date	Results	References
Complex coacervation method	Sustained release, Improve the foliar spread ability of the drug	The cumulative release increased with the increasing pH from 5.0–9.0	NULL	5 October 2017	The chlorpyrifos-loaded microcapsules are of remarkable sustainable-release property. The chlorpyrifos accumulative release rate from PSiSC/NaCMC/GE microcapsules increases with increasing pH.	Dai et al. [29]
Crosslinking, electrostatic interactions and hydrogen bonding between active ester groups	Sustained release	NULL	NULL	December 2017	The drug release mechanism was dominated by a combined effect of drug molecule diffusion and polymer matrix degradation to achieve controlled release. And the release rates decreased with cross-linking degree increased. The drug-loaded composites degraded faster at initial 6 h then slowed down.	Chen et al. [30]
Methods reports by Hao et al. [31]	Good dispersibility and excellent UV resistance	The cumulative release decreased with the increasing pH from 3.0–9.0	NULL	1 March 2020	The thickness of CMC-g-PDMDAAC increased after encapsulated on AVM@P-Zein, meanwhile the anti-UV light performance increased by almost 10% and the adhesion ability increased by approximately 20%. CMC-g-PDMDAAC encapsulated on AVM@P-Zein could lower the sustained release rate of pesticides, in 300 h the release rate slowed down by 10%.	Li et al. [32]
the CS-citral emulsion was slowly dripped into the CMC solution under slow stirring to form microspheres.	Improved fiber adhesion and UV resistance for optimized pesticide loading and intelligent controlled release	NULL	NULL	1 December 2020	The bioactive matrix systems serving dual functions may not only improve the antibacterial activity of CS under neutral pH conditions, but at the same time has the advantages of reducing the loss of volatile organic compounds and oxidation of citral. The best formulation in terms of the ratios of CS, CMC and citral was evaluated, and the highest citral loading ratio reached 68%.	Ma et al. [25]
Interfacial polyaddition in an inverse miniemulsion using toluene diisocyanate (TDI) as a crosslinking agent	degraded selectively by fungal enzymes to release encapsulated agrochemicals	NULL	NULL	11 December 2020	Pyraclostrobin-loaded nanocarriers proved to be active against several pathogenic fungi responsible for devastating plant diseases in horticulture or agriculture. The xylan-based nanocarriers could be degraded by fungi.	Beckers et al. [33]
The hemp hurd, Gelatin, Eugenol and HH were mixed with 15 mL of water and stirred at 45 °C for 4 h.	pH controlled release	The cumulative release decreased with the increasing pH from 3.0–12.0	NULL	22 April 2021	Swelling phenomenon is reduced in acidic conditions while gelatin dissolution rate was slower at basic pH compared to neutral and acidic ones.	Viscusi et al. [34]
Self-assembly	pH controlled release, enhancing the stability of the original drug	The cumulative release increased with the increasing pH from 3.0–9.0	NULL	1 May 2021	AVM encapsulated in AVM@CMC-g-PRSG nanoparticles can be released slowly and last for more than 160 h. AVM@CMC-g-PRSG improve the dispersion and stability of typical insoluble AVM in water and can control the release of drugs by adjusting pH value.	Zhao et al. [35]
HMS-NH_2_ were synthesized by a one-step method, and then the HMS-COOH, HMS-HPC were synthesized by chemical reaction	pH and Cellulase controlled release	The cumulative release decreased with the increasing pH from 3.0–7.0	NULL	15 July 2021	The PYR-HMS-HPC had a PYR loading capacity of approximately 12.1 wt%, quickly released the encapsulated cargo molecule in response to cellulase or a low-pH environment.	Gao et al. [36]

**Table 3:** Nano-intelligent-controlled release carrier system based on starch and dextrin.

Methods	Key Features	pH Sensitivity	Temperature Sensitivity	Publish Date	Results	References
Kneading procedure	Protect the photodegradation of the original drug and sustained release	NULL	NULL	11 March 2014	The formation of pyrimethanil/HP-β-CD inclusion complex increased significantly the photostability of this fungicide in aqueous solutions, presenting a special significance in natural water. Cyclodextrin-encapsulated pyrimethanil was more slowly photodegraded in all the waters. In river water, the fungicide half-lives were increased approximately by a factor of four.	Fernandes et al. [41]
Preparing CMS by method reported elsewhere [42]	Sustained release	NULL	NULL	20 January 2016	The herbicide release rate from CMS/MMT composites in water was significantly reduced when compared to commercial isoproturon—95% released after ca. 700 h and ca. 24 h, respectively.	Wilpiszewska et al. [43]
Casting method	Sustained release	NULL	NULL	6 December 2017	Herbicide loaded in nanotubes displayed much slower release from biodegradable polymer film in water than did free herbicide.	Zhong et al. [40]
Kneading method described by Santos et al. [44]	Protect the photodegradation of the original drug and increase the solubility of the original drug	NULL	NULL	1 February 2018	The nanoformulations presented good colloidal characteristics (size, polydispersity, and zeta potential) and high encapsulation efficiencies for both active agents. The nanocarrier systems may be good candidates for improving the lifetimes of carvacrol and linalool when exposed to the environment.	Campos et al. [45]
Carbonate NSs were prepared by heating a solution of dextrin and CDI in anhydrous DMF	Sustained release	NULL	NULL	June 2021	Dextrin-based nanosponges and γNS-CDI can be suggested as suitable carriers in the formulation of Ail-based herbicide, being able to improve its phytotoxicity and persistence in laboratory under controlled conditions.	Sonia et al. [46]
Extrusion–exogenous gelation method	Sustained release and increase the encapsulation rate	NULL	NULL	January 2022	SCCA formulation exhibited the highest EE and DL and the highest capacity of controlled release of spirotetramat. The degradation rate of spirotetramat in the SCCA formulation was obviously slower than that of spirotetramat in the commercial formulation.	Xie et al. [26]

**Table 4:** Nano-intelligent-controlled release carrier system based on alginic acid.

Methods	Key Features	pH Sensitivity	Temperature Sensitivity	Publish Date	Results	References
mixing a solution containing gelatin, pheromone, and sodium alginate, and dripping it into a CaCl_2_ solution using an injection needle.	Sustained release, extended drug holding period	NULL	NULL	9 August 2008	The release of dodecyl acetate from the beads may be controlled by changes in bead porosity by varying the alginate and gelatin concentrations.	Yosha et al. [50]
Constant stirring	Prevent the original drug light and microbial degradation, sustained release	The cumulative release decreased with the increasing pH from 5.8–11.2	The release of pesticide increased with the increasing temperature from 12 to 27 °C and decreased with temperature from 27 to 35 °C	3 August 2009	The fractional release of the cypermethrin is found to increase with greater percent loading of the pesticide. The extent of release is found to be dependent on the chemical architecture of the bead.	Roy et al. [51]
Ionotropic pre-gelation of polyanion with calcium chloride followed by polycationic crosslinking	Reduce the concentration of the release of Paraquat in the soil	NULL	NULL	15 June 2011	The herbicide showed good association with the alginate/chitosan nanoparticles, which altered its release profile. Sorption tests showed that the soil sorption profile was reduced when paraquat was associated with the nanoparticles, hence improving the herbicidal action.	Silva et al. [7]
Esterification reaction, self-assembled	Controlled release, high encapsulation rate	NULL	NULL	January 2018	A high encapsulation efficiency of acetamiprid in the H-CSAD-CaCl_2_ nanoparticles was achieved, reaching up to 90.8%. The release rate of acetamiprid from the nanoparticles could be controlled by changing the MW of CSAD.	Zhao et al. [52]
Electrostatic action	The resistance and mild release of the plant to the tobacco and lobe virus, sustained release	The cumulative release increased with the increasing pH from 3.0–9.0	The release of pesticide increased with the increasing temperature from 10 to 40 °C	23 August 2019	The AL-hydrogel guaranteed that the antiviral effect of LNT was maintained in the soil for a long time and achieved the sustained release of LNT to induce plant resistance. The formation of the amino-oligosaccharide film further enhanced the slow-release ability of the AL-hydrogel, prolonged the sustained release time, and achieved long-term induction of plant resistance.	Xiang et al. [53]
Stir and mix under temperature control	pH controlled release, sustained release	The cumulative release increased with the increasing pH from 3.0–5.0 and decreased with the increasing pH from 5.0–8.0	NULL	7 October 2019	The Schiff base crosslinking point can provide a gel pH-dependent pesticide releasing ability, whereas the Ca^2+^ crosslinking point can avoid the rapid releasing of the pesticide during environmental pH change. The gel could obviously increase its pesticide releasing rate when the environmental pH was decreased from 7 to 5.	Ma et al. [54]
Electrostatic attraction, self-assembly	Sustained release, water -soluble control conveying system	The cumulative release increased with the increasing pH from 5.0–7.4	NULL	1 November 2020	The propesticide could self-assemble into core-shell structure NPs in aqueous solution. The NPs were capable of releasing pesticide in response to esterase and GSH stimulation and showing high photoactivity.	Xie et al. [55]
Constant stirring, crosslinking	Light controlled release, sustained release	NULL	The release of pesticide increased with the increasing temperature from 25 to 45 °C	August 2021	Based on the volume change caused by the helix-coil transition of gelatin and the photothermal effect of PPy, PPy/Ca-alginate/Gel is sensitive to both temperature and light. It can effectively release the agrochemical contained in it through external photothermal stimulation when using CBZ as a template molecule.	Xing et al. [56]
An inverse miniemulsion template in sunflower oil	Sustained release, extended drug holding period	NULL	NULL	11 September 2021	Alginate nanohydrogels promoted the controlled and sustained release of dicamba over more than two weeks. The nanoformulation of the pesticide slowed down the release rate and exerted a protective action toward hydrophilic active substances.	Artusio et al. [57]

**Table 5:** Nano-intelligent-controlled release carrier system based on guar gum.

Methods	Key Features	pH Sensitivity	Temperature Sensitivity	Publish Date	Results	References
Stir and mix under temperature control	Sustained release	NULL	NULL	8 March 2017	The controlled release study and the kinetics suggest that based on the network properties of the test carriers, the release rate can be manipulated, thus leading to the formation of controlled release systems.	Kumar et al. [68]
Free polymerization under certain temperature and pH conditions	Sustained release and prevents degradation	NULL	NULL	5 January 2022	Higher thermal stability was seen to the loaded PAA-PAAm hydrogels over pure GG and GG-g-PAA-PAAm systems. The GG-g-PAA-PAAm hydrogel system was excellent water holding material in neutral and basic mediums and salt solution.	Karnakar et al. [66]
A solution route abbreviated as “P + H” method	Sustained release	NULL	NULL	3 June 2022	Utilizing the control release system, the polymer–herbicide conjugate gel formulation was prepared and sprayed in the weed nursery of the broad leaf weeds, which increased the retention period of herbicide over the weed leaf surface.	Bhardwaj et al. [67]

## Supplementary Materials

The following supporting information can be downloaded at: https://www.mdpi.com/article/10.3390/polym15132810/s1, Figure S1: Development timeline of chitosan-based nanotechnology intelligent controlled release carrier system; Figure S2: Development timeline of cellulose-based nanotechnology intelligent controlled release carrier system; Figure S3: Development timeline of intelligent controlled release carrier system using starch or dextrin in nanotechnology; Figure S4: Development timeline of intelligent controlled release carrier system using alginic acid in nanotechnology; Figure S5: Development timeline of intelligent controlled release carrier system using Guar gum in nanotechnology; Table S1: Symbol and abbreviations.
