# Supplementary material for: Research Progress of a Pesticide Polymer-Controlled Release System Based on Polysaccharides
Source: Polymers (Basel). 2023 Jun 25;15(13):2810. doi: 10.3390/polym15132810 (PMC10346294; doi:10.3390/polym15132810)
Supplement: Supplementary file 1 [file polymers-15-02810-s001.zip › polymers-2241077-supplementary.pdf]

Supplementary Of Research progress of pesticide polymer-controlled release system based on polysaccharide

Table S1 Symbol and abbreviations

Symbol /Abbreviations	Definitions
Ac-Dex	acetalized dextran
ALA-hydrogel	alginate-lentinan-amino-oligosaccharide hydrogel
AL-hydrogel	alginate-lentinan drug-loaded hydrogel
AVM	Avermectin
BTDA	3,3′,4,4′-benzophenonetetracarboxylic dianhydride
Ca-alginate	alginate calcium
CBZ	Carbendazim
CdS	cadmium sulfide
cl-Ch-pMAc	poly (methacrylic acid) crosslinked chitosan
CMC	carboxymethyl cellulose
CMC-g-PDMDAAC	graft copolymer by electrostatic interaction, in which carboxymethyl cellulose and diallyldimethylammonium chloride as monomers
CMC-g-PRSG	nanocarriers are prepared from two compounds from natural materials from natural materials carboxymethyl cellulose (CMC) and rosin (RS)
CMS	Carboxymethyl starch
CO	Chitosan oligomer
CRR	cumulative release rate
CS	chitosan
CSAD	cholesteryl-grafted sodium alginate derivatives
CVC	carvacrol
DCMC	N,N-dimethylhexadecyl carboxymethyl chitosan
DIN	dinotefuran
DL	drug loading
DMDAAC	diallyldimethylammonium chloride
DMF	N,N-dimethylformamide
EE	entrapment efficiency
EVA	poly(ethylene-*co*-vinyl acetate)
GC film	film based on guar gum incorporated with citral
GE	gelatin
GEL/Gel	gelatin
GG	guar gum
GG-g-PAA-PAAm	guar gum grafted acrylic acid – acrylamide
GG-HG	guar gum-g-cl-polyacrylate/bentonite hydrogel composite
GG-NHG	guar gum-g-cl-PNIPAm nanohydrogel
GSH	Glutathione
H-CSAD-$CaCl_2$	High molecular weight calcium chloride cholesterol grafted sodium alginate derivative
HH	Hemp Hurd

HMS	Hollow mesoporous silica
HPC	Hydroxypropyl cellulose
HP-β-CD	Hydroxypropyl--Cyclodextrin
ID	Iprodione
IPP	poly(ethylene glycol)-poly(ε-caprolactone)
LNL	linalool
LNT	Lentinan
MCN	Relevant quantity of MC, NHS, EDC was weighed by the ratio of 1:2:4 and dissolved in distilled water. After gently stirred for 1 h at 25 °C, the solid was vacuum filtered, washed with distilled water several times and dried under vacuum at 50 °C to get purified MCN
MDI	methane-4,4′-diisocyanate
MMT	montmorillonite
MW	molecular weight
NaCMC	sodium carboxymethylcellulose
NCT	nicotine hydrochloride
NH_2	Amino
NPs	nanoparticles
NSs	nanosponges
PAA-PAAm	polyacrylic acid-polyacrylamide
PCL	poly(ε-caprolactone)
PEC	pectin
PEG	poly(ethylene glycol)
PPy	polypyrrole
PSiSC	polysiloxane sodium carboxylate
PYR	Pyraclostrobin
P-Zein	phosphorylated zein
QDs	quantum dots
SA	sodium alginate
SCCA	starch–chitosan–calcium alginate
TDI	toluene diisocyanate
TMV	tobacco mosaic virus
TPP	tripolyphosphate
UV	Ultraviolet
ZnO	Zinc oxide
γNS-CDI	dextrin-based nanosponges synthesized with 1,1′-carbonyldiimidazole
γ-PGA	poly-γ-glutamic acid

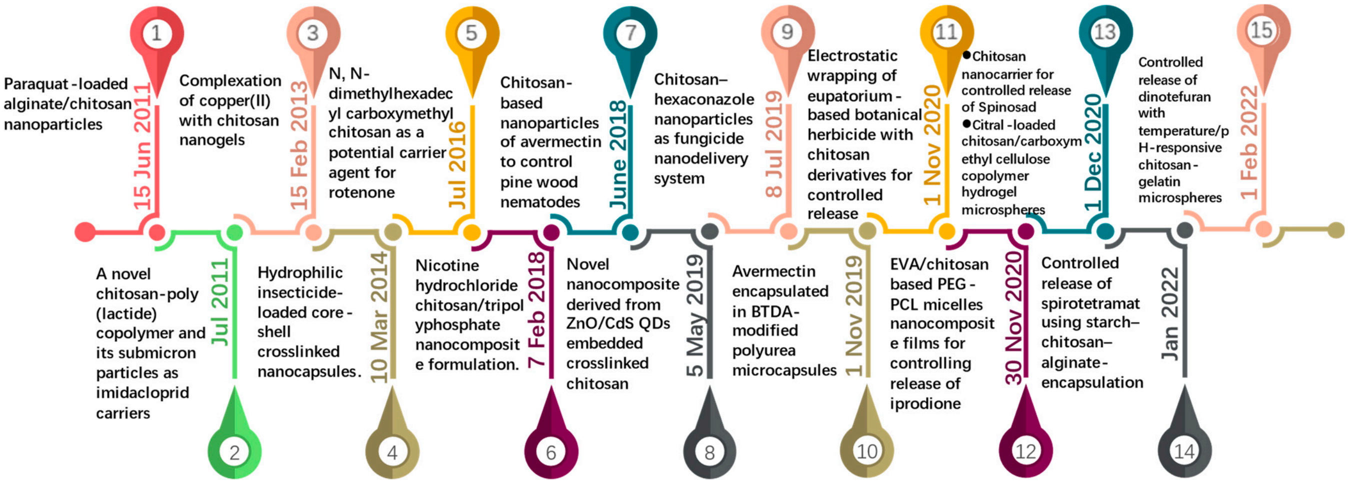

Figure S1 Development timeline of chitosan-based nanotechnology intelligent controlled release carrier system

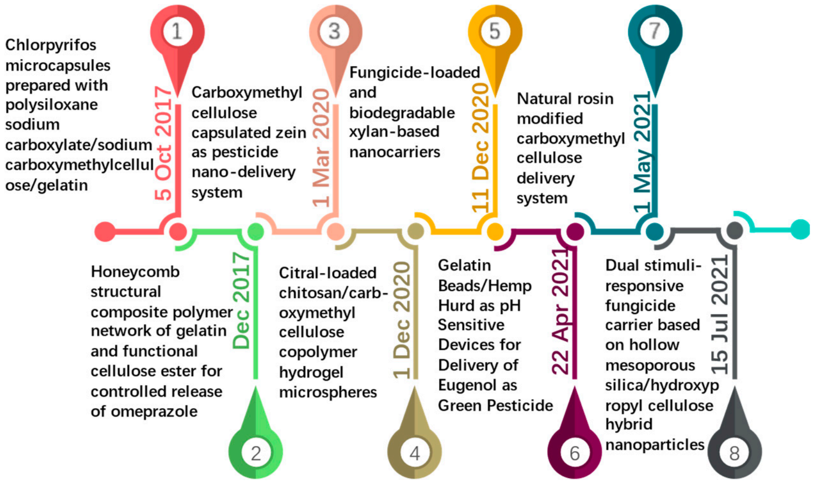

Figure S2 Development timeline of cellulose-based nanotechnology intelligent controlled release carrier system

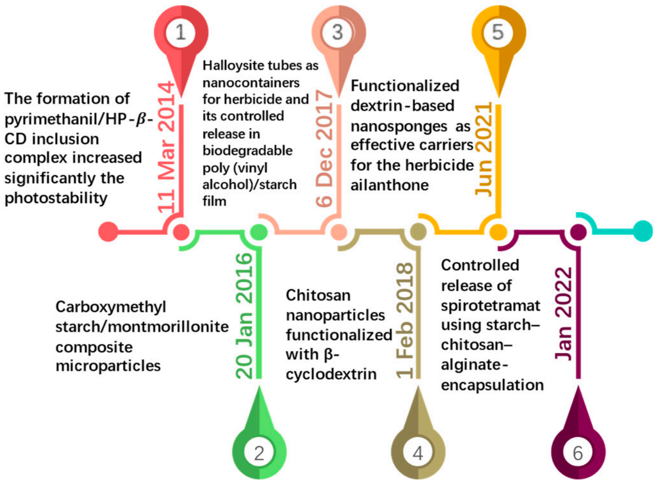

Figure S3 Development timeline of intelligent controlled release carrier system using starch or dextrin in nanotechnology.

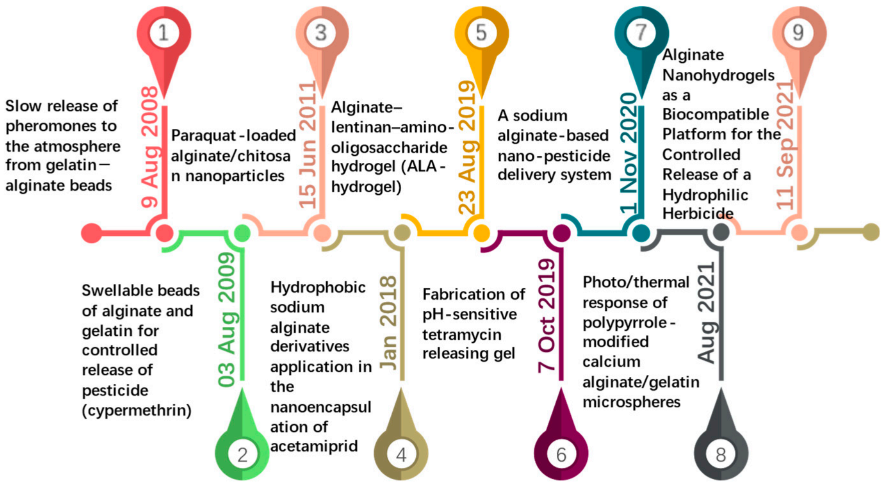

Figure S4 Development timeline of intelligent controlled release carrier system using alginic acid in nanotechnology.

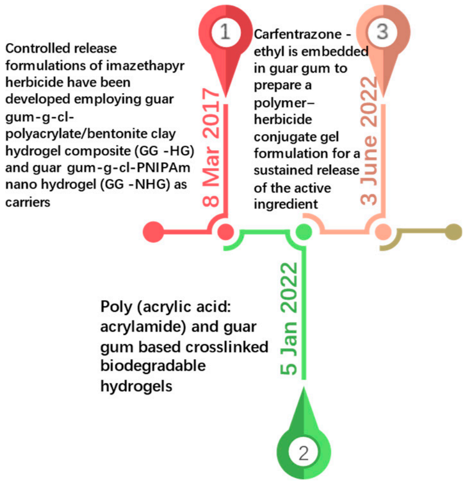

Figure S5 Development timeline of intelligent controlled release carrier system using Guar gum in nanotechnology.

`
